# Conflictual influence of humidity during shelter selection of the American cockroach (*Periplaneta americana*)

**DOI:** 10.1038/s41598-019-56504-w

**Published:** 2019-12-30

**Authors:** Mariano Calvo Martín, Stamatios C. Nicolis, Isaac Planas-Sitjà, Jean-Louis Deneubourg

**Affiliations:** 1 Biological and Artificial Self-organised Systems Team, Université Libre de Bruxelles, Brussels, Belgium; 2 Evolutionary Biology & Ecology Unit, Université Libre de Bruxelles, Brussels, Belgium; 3 Systematic Zoology Laboratory, Tokyo Metropolitan University, 1-1 Minami-Osawa, Hachioji Tokyo, 192-0397 Japan

**Keywords:** Behavioural ecology, Animal behaviour

## Abstract

In collective decision-making, when confronted with different options, groups usually show a more marked preference for one of the options than do isolated individuals. This results from the amplification of individual preferences by social interactions within the group. We show, in an unusual counter-example, that when facing a binary choice between shelters with different relative humidities, isolated cockroaches of the species *Periplaneta americana* select the wettest shelter, while groups select the driest one. This inversion of selection results from a conflictual influence of humidity on the probabilities of entering and leaving each shelter. It is shown that the individual probability of entering the wettest shelter is higher than the group probability and is increased by previous entries and exits. The probability of leaving each shelter decreases in the population due to social interactions, but this decrease is less pronounced in the wettest shelter, suggesting weaker social interactions. A theoretical model is developed and highlights the existence of tipping points dependent on population size, beyond which an inversion of selection of a resting place is observed.

## Introduction

Social animals are able to collectively choose the most suitable resource among several options through social interactions (e.g., individual interattractions, communication^1^), maintaining in this way the cohesion of the group and subsequent social benefits^2^. In other words, they express a preference for a resource. These choices and preferences are modulated by different factors, such as physiological factors (e.g., desiccation), environmental factors (e.g., temperature, humidity^3^), or sociality-related factors (e.g., positive and/or negative interactions, group size^4,5^).

Terrestrial arthropods are highly sensitive to variations in humidity and subsequent water loss, which occur mainly through evapotranspiration and respiration^6,7^. Extreme conditions can severely impact the well-being of animals. Dry environments lead to an increased rate of water loss, which subsequently results in the dehydration of the animal. On the other hand, at high levels of humidity, the cooling effect of evapotranspiration is nullified^7^, overheating the animal. This highlights the importance of an adequate water balance as essential to maintaining homeostasis in terrestrial arthropods. To do so, most arthropods benefit from different mechanisms to counterbalance water loss^8^. These mechanisms can be physiological, by modifying the water content of their faeces^9^ or their respiration rate^10^, They can also be behavioural, such as hygrotaxis to acquire environmental moisture^11^ through fog basking^12^ or forming an aggregate to reduce individual water loss^13–16^ mainly by reducing the individual surface area exposed to the air^17^.

Many studies on gregarious arthropods and eusocial insects^18^ have shown that the presence of conspecifics is able to amplify individual preferences through positive social interactions^2,19^, leading to better discrimination of the quality of resources^20–22^. In social and gregarious insects, these social interactions often involve cuticular hydrocarbons^1,23^. This classical approach of collective decision-making often underestimates the modulation of the strength of social interactions induced by the environment^15,24,25^. Indeed, it has been reported that large groups of individuals show different preferences from those of smaller groups or isolated individuals, such as bark beetles (Scolytinae) attacking trees^26^ or spiny lobsters selecting shelters^27^, but the origin of these different preferences seems to be related to crowding effects. Recently, a study on the American cockroach showed a crowding-independent inversion of scented shelter selection between isolated individuals and groups^28^, indicating that this type of phenomenon could also be at work in other situations.

Here, we test the selection of a resting place between two shelters with different relative humidity (RH) levels offered to both isolated individuals (isolates) and groups of the American cockroach *Periplaneta americana* (L.). The hypothesis put forward in this paper is that isolated individuals would prefer a shelter with high RH (i.e. a wet shelter, hereafter WS) as a resting place to avoid water loss^29,30^. In contrast, a group of cockroaches should select a drier shelter (hereafter DS) as a resting place, as high levels of humidity reduce the strength of social interactions^15,24^.

## Results

### Sheltered individuals

**Figure 1:**
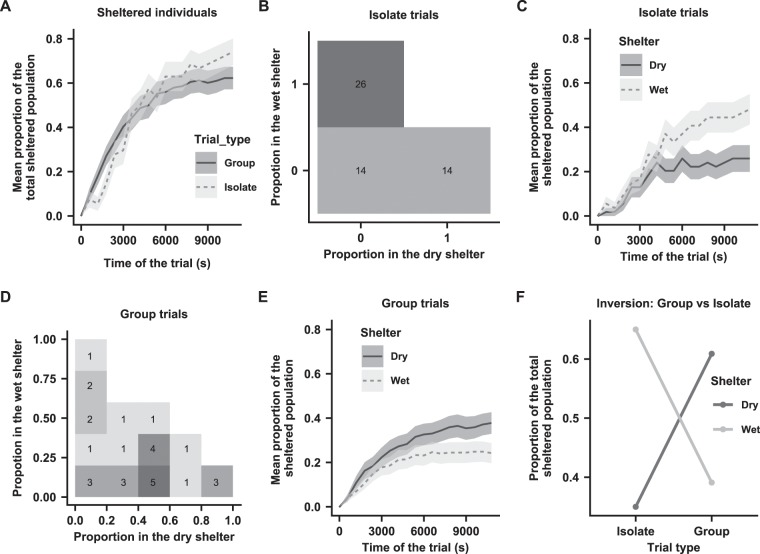
(**A**) Mean and standard error of the proportion of sheltered individuals over time (*s*) in the isolate (light grey) and group (dark grey) trials. (**B**) Two-dimensional histogram of the total proportion of the isolated individuals in the DS and WS at the end of the experiment. (**C**) Mean and standard error of the proportion of sheltered individuals over time (*s*) inside the WS (light grey) and the DS (dark grey) for the isolate trials. (**D**) Two-dimensional histogram of the total proportions of the groups in the DS and WS at the end of the experiment. (**E**) Mean and standard error of the proportion of sheltered individuals over time (*s*) inside the WS (light grey) and the DS (dark grey) for the group trials. (**F**) Proportion of all individuals choosing the DS (dark grey) and WS (light grey) at the end of the trials for the isolated individuals and the groups.

The size of the total sheltered population increased over time (Table 1 and Fig. 1A), and the proportion of individuals sheltered at the end of the experiments (each trial lasted 10800 s) was not significantly different between the isolated individuals (0.74) and the groups (0.63) trials $({\chi }_{{1}_{(N=518)}}^{2}\,=\,2.9,\,{\rm{P}}=0.09)$. Regarding shelter selection, isolated individuals (Fig. 1B) settled more frequently under the WS than under the DS (0.65 of the total sheltered population, permutation test: P = 0.04, at the end of the trials (3 *h*)), while groups (Fig. 1D) selected the DS more frequently (0.61 of the total sheltered population, permutation test: P = 0.004, at the end of the trials). The selection of one shelter over the other occurred at least 6000 s after the beginning of the trials (Fig. 1C,E). After when the population inside the shelters was stabilized. This inversion of shelter selection between the two conditions was significant (Fig. 1F; ${\chi }_{{1}_{(N=329)}}^{2}\,=\,8.628,\,{\rm{P}}=0.0033$). Moreover, most of the individuals/groups that reached a decision at the end of the trials were stable for at least 5400 s before the end, as shown by the plateau in Fig. 1A and Figure S1A,B). In groups, the observed variance between replicates (17.78) for the total sheltered population, at the end of the trials, was significantly higher than the theoretical variance expected under a binomial distribution (P < 0.001; see Material and methods). This indicates, that social interactions were at work and therefore that individual choices were not independent. Finally, a permutation test showed early selection of the DS by the groups as a preferred resting place. Additionally, early selection of the WS by the isolated individuals was shown (see Figure S1A,B).

**Table 1:** Variables measured for the isolate and group trials.

Variables	Both	Wet	Dry
**Isolate (N = 54)**			
N entries (overall replicates)	283	170	113
Mean N entries (±SD)	5.77 (±4.87)	3.14 (±2.89)	2.09 (±2.74)
Total time	280371.2 s	173213 *s*	107158.2 *s*
Mean total time (±SD)	5192 *s* (±3768.1 *s*)	3208.6 *s* (±3716 *s*)	1984.4 (±3725.5 *s*)
Mean visit time (±SD)	990.7 *s* (±24165.5 *s*)	962.2 *s* (±2369.1 *s*)	830.6 *s* (±2277.8 *s*)
N wins (proportion)	— (−)	26 (0.48)	14 (0.26)
**Group** ***(N***_*exp*_ **= 29)** **(N**_***ind***_ **= 464)**			
N sheltered at 3 h (proportion)	289 (0.62)	113 (0.24)	176 (0.37)
Mean sheltered ind. at 3 h (±SD)	10.04 (±4.37)	3.98 (±3.91)	6.06 (±4.21)
N entries (overall replicates)	1841	990	851
Mean N entries (±SD)	63.54 (±29.93)	34.13 (±18.99)	29.41 (±17.68)
Total time	424320 *s*	202619 *s*	221691 *s*
Mean total time (±SD)	14629.9 *s* (±5732.5 *s*)	6986.8 *s* (±3808.3 *s*)	7644.5 *s* (±3609.8 *s*)
N wins (proportion)	— (−)	8 (0.28)	20 (0.69)

### Entries

The mean numbers of individual entries were similar between the isolate and the group trials (means ± SD - isolates: 5.77 ± 4.8 - groups: 3.96 ± 1.87; Wilcoxon test: W = 730.5, P = 0.62). However, the number of entries in all experiments was higher for the WS than for the DS (Table 1; isolates ${\chi }_{{1}_{(N=54)}}^{2}=11.044$, P < 0.001; groups ${\chi }_{{1}_{(N=29)}}^{2}=10.052$, P = 0.001). A permutation test confirmed these differences (isolates: P=0.01; groups: P < 0.001). The proportions of entries of the WS in the isolate and group trials were 0.6 and 0.54, respectively, and were not significantly different (Wilcoxon test: W = 564.5, P = 0.13).

### Influence of RH on leaving rates

**Figure 2:**
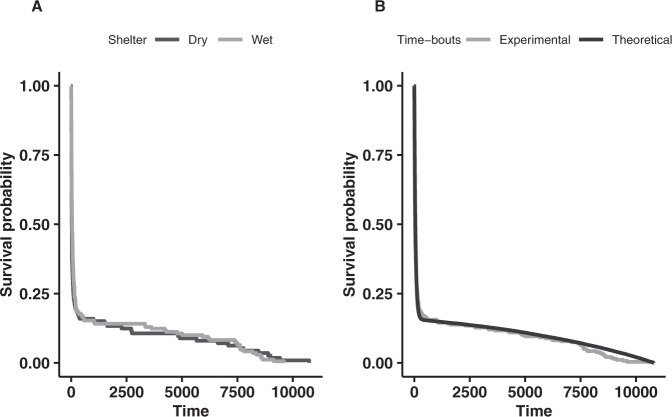
(**A**) Survival curve of the time-bouts (*s*) in the DS (dark grey) and WS (light grey) for the isolated individuals. (**B**) Survival curve of the time-bouts (*s*) under the shelters for the experimental (isolate) trials (light grey) and the theoretical simulation (black) for the isolated individuals.

Regarding the isolate trials, the mean (± SD) time-bouts in the WS and DS (Table 1) were not significantly different (Wilcoxon test: W = 8298.5, P = 0.053). Moreover, a survival analysis of the time-bouts of the isolated individuals (Fig. 2A) showed no significant difference between the WS and DS (log-rank test: ${\chi }_{1}^{2}=0.98$, P = 0.32). This analysis highlights the existence of at least two time-bout regimes, with a large majority (84.5%) of short stays and a minority of longer stays, because the regression could not be fitted by a single exponential curve. Finally, the ratio between entries and exits per trial (Wilcoxon test: W = 1733, P = 0.08) showed no significant differences between the WS and DS. This result is in agreement with a higher frequency of entering the WS and comparable resting times in the two types of shelters. For the group trials, this ratio was larger for the DS (Wilcoxon test: W = 237.5, P = 0.002), which means that the number of exits of the DS was smaller than that of the WS. Combined with the fact that the number of entries was smaller in the DS (see Entries), this indicates that cockroaches more frequently left the WS.

### Mechanisms of entering and leaving the shelter

#### Entering a shelter

Cockroaches outside the shelters have a global probability to enter a shelters per unit time estimated as the inverse of the mean time-bout outside the shelters, in the isolate trials. Let *J*_*d*_ (*t*) and *J*_*w*_ (*t*) be the entering probabilities per unit time (*s*^−1^) of the DS and WS, respectively. The relative probabilities of entering the DS (WS) at time *t* can be written as:

1 $${P}_{d}(t)=\frac{{J}_{d}(t)}{{J}_{d}(t)+{J}_{w}(t)}\,{P}_{w}(t)=1-{P}_{d}(t)$$

Two hypotheses can be formulated for *J*_*d*_ (*t*) and *J*_*w*_ (*t*):

1. Entering the DS (WS) depends only on the intrinsic attractivity of the shelters. The relative entering probability (*P*_*d*_; *P*_*w*_) is thus constant and equal to the corresponding proportion of the total number of entries:
  2 $${P}_{d}=\frac{{F}_{d}}{{F}_{d}+{F}_{w}}\,{P}_{w}=1-{P}_{d}=\frac{{F}_{w}}{{F}_{d}+{F}_{w}}$$
  where *F*_*d*_ and *F*_*w*_ are the total numbers of entries of the DS and WS during the experiment, respectively. For the isolated individuals, *P*_*d*_ and *P*_*w*_ are equal to 0.4 and 0.6, respectively, and for the groups, they are 0.46 and 0.54 (see Entries). With this hypothesis, the number of entries of the DS and WS would follow a binomial distribution with a probability of success equal to *P*_*d*_ and *P*_*w*_, respectively. A binomial test for the isolated individuals showed that the results of 52 of 54 experiments were inside the 95% confidence interval (CI). The same test for the groups showed, however, that only 14 of 29 experimental observations were inside the 95% CI, which contradicts the hypothesis of constant entering probabilities.
2. An alternative hypothesis is that cuticular hydrocarbon deposition (actively or passively) at the shelter entrances influences future decisions to enter the shelters^31^. In particular, cockroaches can select a resting place based only on hydrocarbon deposition, even if the site is empty^32^. Thus, the probability of entering a shelter will depend on both the number of previous events (entries and exits) and the intrinsic attractivity of the shelter. This statement is supported by significant dependence of the sign of the difference of past events between DS and WS before an entry of the DS (or WS) (isolate trials: ${\chi }_{{2}_{(N=526)}}^{2}=7.4282$, P = 0.0024; group trials: ${\chi }_{{2}_{(N=1840)}}^{2}=92.0467$, P < 0.0001; global (isolate plus group trials): ${\chi }_{{2}_{(N=2366)}}^{2}=97.214$, P < 0.0001). A generic function expressing the positive relationship between the probability of entering a shelter and previous marking at its entrance is

3 $${J}_{d}(t)=k{\alpha }_{d}{e}^{\zeta {M}_{d}}\,{J}_{w}(t)=k{\alpha }_{w}{e}^{\zeta {M}_{w}}$$

where *k* and *α*_*d*_ (*α*_*w*_) are the global probability of entering a shelter and the intrinsic attractivity of the DS (WS), respectively. *M*_*d*_ (*M*_*w*_) is the sum of the past entries *G*_*d*_ (*G*_*w*_) and exits *S*_*d*_ (*S*_*w*_) of the DS (WS):

4 $${M}_{d}=\mathop{\sum }\limits_{i=0}^{t-1}{G}_{{d}_{i}}+{S}_{{d}_{i}}\,{M}_{w}=\mathop{\sum }\limits_{i=0}^{t-1}{G}_{{w}_{i}}+{S}_{{w}_{i}}$$

*ζ* accounts for the influence of the hydrocarbons deposited at the entrance. Combining Eq. 3 into Eq. 1 yields

5 $${P}_{d}(t)=\frac{1}{1+{\alpha }_{w/d}{e}^{-\zeta ({M}_{d}-{M}_{w})}}\,{\rm{w}}{\rm{i}}{\rm{t}}{\rm{h}}\,{\alpha }_{w/d}=\frac{{\alpha }_{w}}{{\alpha }_{d}}$$

Equation 5 is the individual relative probability of entering the DS and is valid for both isolated individuals and individuals in groups. Parameters *α*_*w/d*_ and *ζ* were estimated by adjusting Eq. 5 based on the combined number of entries in isolate and group trials via a nonlinear least square model using the Levenberg-Marquardt algorithm^33^ (*M*_*d*_–*M*_*w*_ were binned by intervals of 10 (n = 3918, residual standard error = 0.08155)), with *ζ* = 0.0133 ± 0.002 (P < 0.0001), *α*_*w/d*_ = 1.28 ± 0.11, *α*_*d*_ = 0.44. ± 0.11, and *α*_*w*_ = 0.56. ± 0.11 (P < 0.0001).

#### Leaving the shelter

##### Influence of RH

The distribution of the time-bouts (see section Influence of RH on leaving rates) is in agreement with that of Jeanson and Deneubourg^34^. This could be the result of two different probabilities of leaving the shelters. In other words, an individual that enter a particular shelter can remain in an excited state, with a high leaving probability *L*_*e*_, or switch to a calm state, with a low leaving probability *L*_*c*_. The resulting proportions of individuals in excited and calm states are expressed as *Q* and 1–*Q*, respectively. *L*_*e*_, *L*_*c*_ and *Q* were estimated by numerical optimisation using a simulation (10000 realisations) of the time-bouts of isolated individuals. For the time-bouts, no difference was observed between the DS and WS (see Influence of RH on leaving rates); therefore, we combined the time-bouts in both shelters. The best estimated probabilities were *L*_*e*_ = 0.014 *s*^−1^, *L*_*c*_ = 0.000031 *s*^−1^ and *Q* = 0.8. The comparison of the survival curves of time-bouts revealed no difference between the theoretical and experimental curves (Fig. 2B, log-rank test: ${\chi }_{1}^{2}=1.3$, P = 0.2).

##### Interplay between the influence of conspecifics and RH on the leaving probability

In group trials, the decision to leave the shelter (DS or WS) was not synchronized among individuals. Based on the analysis of the isolated individuals and previous studies showing that leaving probabilities decrease with the number of individuals present in the shelter^21^, our hypothesis regarding leaving probabilities is that cockroaches entering a shelter remain in an excited state or adopt a calm state, with *Q* probability of remaining in the excited state, and that these probabilities are the same for the DS and WS. The number of settled individuals in the excited state *E*_*d*_ (*E*_*w*_) and in the calm state *D*_*d*_ (*C*_*w*_) under the DS (WS) can be written as

6 $$\begin{array}{cc}{E}_{d}=Q{i}_{d} & {E}_{w}=Q{i}_{w}\\ {C}_{d}=(1-Q){i}_{d} & {C}_{w}=(1-Q){i}_{w}\end{array}$$

where the sheltered individuals *i*_*d*_ (*i*_*w*_) under the DS (WS) have a probability of leaving their shelter in an excited state *ρ*_de_ (*ρ*_we_) or in a calm state *ρ*_dc_ (*ρ*_wc_), which decreases with the sheltered population

7 $$\begin{array}{c}{\rho }_{de}={{L}^{{\rm{^{\prime} }}}}_{e}{e}^{-{\gamma }_{d}({i}_{d}-1)}+{\beta }_{de}\,{\rho }_{we}={{L}^{{\rm{^{\prime} }}}}_{e}{e}^{-{\gamma }_{w}({i}_{w}-1)}+{\beta }_{we}\\ {\rho }_{dc}={{L}^{{\rm{^{\prime} }}}}_{c}{e}^{-{\gamma }_{d}({i}_{d}-1)}+{\beta }_{dc}\,{\rho }_{wc}={{L}^{{\rm{^{\prime} }}}}_{c}{e}^{-{\gamma }_{w}({i}_{w}-1)}+{\beta }_{wc}\end{array}$$

where *γ*_*d*_ (*γ*_*w*_) is the strength of social interactions between conspecifics. ${L^{\prime} }_{e}$ and ${L^{\prime} }_{c}$ refer, respectively, to the excited and calm state leaving rates from a shelter, and *β*_*de*_ (*β*_*we*_) and *β*_*dc*_ (*β*_*wc*_) are the basal exit rates from the DS (WS) in the excited and calm states. ${L^{\prime} }_{e}+{\beta }_{de}$, ${L^{\prime} }_{e}+{\beta }_{we}$, ${L^{\prime} }_{c}+{\beta }_{dc}$ and ${L^{\prime} }_{c}+{\beta }_{wc}$ correspond to the leaving rates of the individuals in the excited and calm states. The mean leaving probabilities from the DS and WS with *i* settled individuals are:

8 $$\begin{array}{c}{\Theta }_{d}({i}_{d})={\varepsilon }_{d}\,{e}^{-{\gamma }_{d}({i}_{d}-1)}+{\beta }_{d}\,{\Theta }_{w}({i}_{w})={\varepsilon }_{w}\,{e}^{-{\gamma }_{w}({i}_{w}-1)}+{\beta }_{w}\end{array}$$

where

9 $$\begin{array}{c}{\varepsilon }_{d}=\frac{({{L}^{{\rm{^{\prime} }}}}_{e}{E}_{d}+{{L}^{{\rm{^{\prime} }}}}_{c}{C}_{d})}{{i}_{d}}\,{\beta }_{d}=\frac{({\beta }_{de}{E}_{d}+{\beta }_{dc}{C}_{d})}{{i}_{d}}\\ {\varepsilon }_{w}=\frac{({{L}^{{\rm{^{\prime} }}}}_{e}{E}_{w}+{{L}^{{\rm{^{\prime} }}}}_{c}{C}_{w})}{{i}_{w}}\,{\beta }_{w}=\frac{({\beta }_{we}{E}_{w}+{\beta }_{wc}{C}_{w})}{{i}_{w}}\end{array}$$

As *E*_*d*_ (*E*_*w*_) and *C*_*d*_ (*C*_*w*_) are assumed to be proportional to *i*_*d*_ (*i*_*w*_) (see Eq. 6), *ε* and *β* become constant. The number of events *T*_*d*_ (*T*_*w*_) where an individual leaves the DS (WS) is proportional to the number of time steps *O*_*d*_ (*i*_*d*_) (*O*_*w*_ (*i*_*w*_)) where *i* individuals are settled in the DS (WS) (*i* = 0, 1, …, 16):

10 $${T}_{d}({i}_{d})={\Theta }_{d}({i}_{d}){i}_{d}{O}_{d}({i}_{d})\,{T}_{w}({i}_{w})={\Theta }_{w}({i}_{w}){i}_{w}{O}_{w}({i}_{w})$$

or

11 $${\Theta }_{d}({i}_{d})=\frac{{T}_{d}({i}_{d})}{{i}_{d}{O}_{d}({i}_{d})}\,{\Theta }_{w}({i}_{w})=\frac{{T}_{w}({i}_{w})}{{i}_{w}{O}_{w}({i}_{w})}$$

Nonlinear least square fitting of ${\Theta }_{d}({i}_{d})$ and ${\Theta }_{w}({i}_{w})$ for the sheltered population (0 to 16 individuals) in each shelter (DS and WS) was undertaken (DS: residual standard error = 5.19 × 10–5; WS: residual standard error = 3.05 × 10–7). The fitted parameters are summarised in Table 2. The CIs of the estimated *ε*’s and *β*’s for DS and WS overlap, indicating that these parameters were similar for both shelters. The results for the isolate trials are in agreement with the absence of a difference in time-bouts between the shelters (see Influence of RH on leaving rates). However, the CIs of the strength of social interactions under the DS (*γ*_*d*_ = 1.1, P < 0.0001) and under the WS (*γ*_*w*_ = 0.7, P < 0.0001) did not overlap and are therefore assumed to be different (*γ*_*d*_ > *γ*_*w*_). This assumption is supported by Dambach and Goehlen^15^, where the authors showed that social interactions decreased with high levels of RH.

**Table 2:** Summary of the parameter values fitted from Eq. 11.

Parameter	Estimated	2.5% CI	97.5% CI
**Dry shelter**			
*∈*_*d*_	5.8 × 10^−3^	5.2 × 10^−3^	6.3 × 10^−3^
Strength of social interaction *γ*_*d*_	1.1051	0.8756	1.3347
Basal leaving probability *β*_*d*_	3 × 10^−3^	2 × 10^−3^	5 × 10^−3^
**Wet shelter**			
*∈*_*w*_	5.8 × 10^−3^	5.3 × 10^−3^	6.1 × 10^−3^
Strength of social interaction *γ*_*w*_	0.7234	0.6206	0.8262
Basal leaving probability *β*_*w*_	3 × 10^−3^	2 × 10^−3^	4 × 10^−3^

## Model

**Figure 3:**
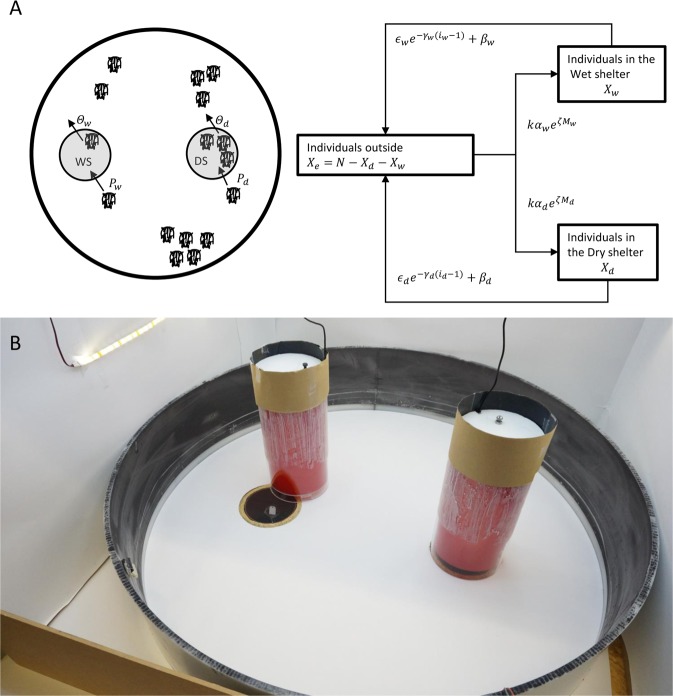
(**A**) Experimental setup. (**B**) Schema of the transitions and their respective probabilities.

We incorporated the mechanisms highlighted in the previous section in a stochastic simulation of the sheltering process (see Fig. 3A for a summary of the main steps of the simulation). At the initial state (*t* = 0), all individuals are located outside (*i*_*d*_ = 0, *i*_*w*_ = 0). At each time step, individuals located outside the shelters have a global probability of entering the DS or WS equal to the inverse of the mean time-bout before an entry of the isolated individuals. Then, individuals choose the DS (or WS) with a probability defined in Eq. 5. Once inside a shelter, individuals are in an excited state and have a probability of becoming calm. They then have a probability of leaving the shelter, as defined in Eq. 11. Note that *β*_*d*_ and *β*_*w*_ were not taken into account because their values were negligible. Furthermore, as Eq. 11 does not consider the proportions of excited and calmed individuals varying through time it is not surprising that the fitted parameter values (see Table 2) as obtained from the leaving rates for the group trials (see Figure S2) are not the ones that give the best fit between the experimental results and the simulation. Instead, *γ*_*d*_ and *γ*_*w*_ used in the simulation were 1.3 and 0.7 respectively (and not 1.1 and 0.72), these values being still inside the CI from the estimated values (see Table 2). Moreover, because of the formation of small clusters outside the shelter (retaining individuals to enter a shelter), we implemented in the simulation a probability of individuals participating in the process equal to 2/3. The simulation was run for 10800 time steps (corresponding to the three hours of the experiment) and 50000 realisations. We sampled sets of trials from the simulation for both the isolated individuals (10000 × 54) and the group (10000 × 29) to compare the distributions of the total sheltered individuals and sheltered individuals in the DS from our model to the experimental data. The agreement between the theoretical and experimental results is shown in Table 3 and validates the hypotheses of the model.

**Table 3:** Comparison between simulated and experimental results.

	Simulation	Experiments
	Mean ± SD	Observed
**Isolated individuals**		
Total sheltered	38 ± 3	40
Dry shelter	16 ± 3	14
**Groups**		
Total sheltered	295 ± 12	289
Dry shelter	168 ± 21	176

**Figure 4:**
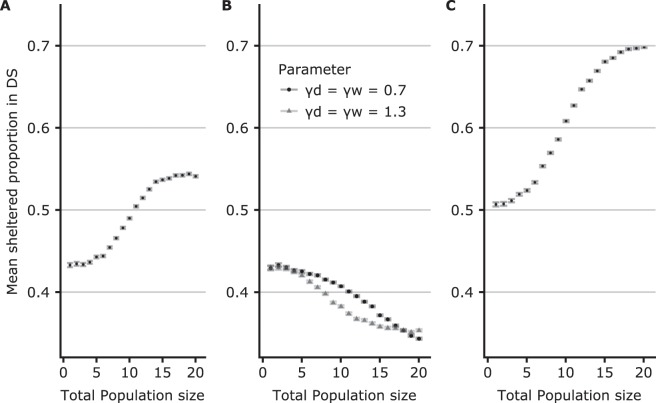
(**A**–**D**) Mean simulated (50000 realisations) proportions of sheltered individuals in the DS defined by Eqs. 5–11. (**A**) Experimental parameter values (*α*_*d*_ = 0.43; *γ*_*d*_ = 1.3; *γ*_*w*_ = 0.7). (**B**) Same strengths of social interactions between the shelters: Black dots (*α*_*a*_ = 0.43; *γ*_*d*_ = 1.3; *γ*_*w*_ = 1.3); Grey triangles (*α*_*d*_ = 0.43; *γ*_*d*_ = 0.7; *γ*_*w*_ = 0.7). (**C**) Same individual shelter preferences for a shelter (*α*_*d*_ = 0.5; *γ*_*d*_ = 1.3; *γ*_*w*_ = 0.7). Other experimental parameter values: *ζ* = 0.0133; *L*_*e*_ = 0.014; *L*_*c*_ = 0.000031.

**Figure 5:**
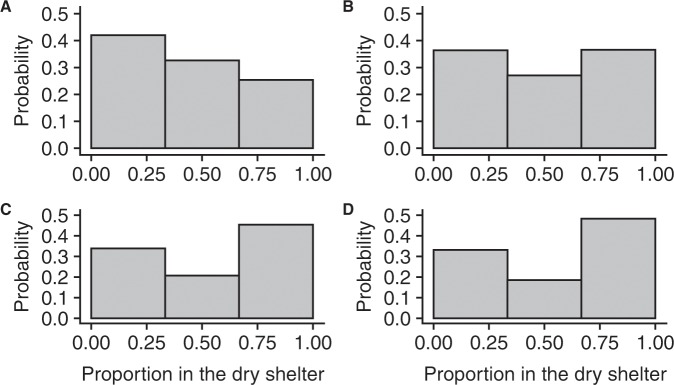
(**A–D**) Probability histograms of the proportion of the total sheltered individuals in the DS based on 50000 realisations of the model at different population sizes. (**A**) Population size = 8. (**B**) Population size = 12. (**C**) Population size = 16. (**D**) Population size = 20. Experimental parameter values are as in Fig. 4A.

We also performed simulations to highlight the role of the key parameters. Figure 4A shows the dependence of population size on selection of the DS with parameter values fitted from the experiments. As seen, at a small population size (1 to 6 individuals), shelter selection favours the WS and therefore depends principally on individual attraction towards the WS. As the population size increases, social interactions in the shelter begin to play a more important role, reducing the probability of selecting the WS. This finding confirms that the interplay between less strong social interactions in the WS and a higher probability of entering this shelter is at the origin of the inversion of selection. In other words, keeping the intrinsic attractiveness of the DS and WS of the experiments (*α*_*w*_ > *α*_*d*_) but changing the rules of social interactions by making them equal (*γ*_*w*_ = *γ*_*d*_) would prevent this inversion, leading to the classical mechanism of amplification of individual preferences by the group. The strength of amplification depends on the value of *γ* and on population size (Fig. 4B). Finally, Fig. 4C also shows a situation where the DS and WS have the same intrinsic attractiveness (*α*_*w*_ = *α*_*d*_) but with the experimental strength of social interactions in the WS and in the DS (*γ*_*w*_ < *γ*_*d*_). Here, an isolated individual selects the WS or DS with equal probability, and as the total population size increases, the mean number of sheltered individuals in the DS increases. Finally, for all the parameters tested (Fig. 4), the system displays multistability. For example, as the group size increases (for the experimental parameter values, see Fig. 5A,D), the mean proportion of sheltered individuals in the DS increases due to a more frequent consensus to settle in the DS.

## Discussion

Our results confirm the proclivity of cockroaches for selecting a dark resting place (a shelter^35^). We also showed that isolated individuals have a clear preference to select the shelters with high humidity as a resting place. However, this was not observed in the group trials, where over all experiences, the average population under the DS was larger than that under the WS. However, this system displays multistationarity: some trials ended with the selection of the WS. This pattern of aggregation cannot be explained without social interactions between individuals, involving components emitted by individuals, mainly cuticular hydrocarbons^36,37^.

Regarding the mechanisms of entering a shelter, we showed a difference in the number of entries between the DS and WS for both the isolate and the group trials (Table 1), showing that RH has an attractant effect on cockroaches. The tendency to enter a shelter also depends on the previous events (entries of and exits from the shelter). This may be the result of the deposition of hydrocarbons at the entrance of the shelters. Indeed, it has been shown^36^ that deposited hydrocarbons can induce the selection of a resting place in the American cockroach. Not surprisingly, there was a significant dependence of the sheltered population size in the DS on the difference in past events (entries and exits) (Spearman correlation = 0.52, P < 0.0001). Therefore, we may assume that the emission of pheromones increases with the sheltered population, which in turn increases the entering probability. Nonetheless, the pheromones of aggregation (for the cockroaches) are mainly heavy hydrocarbons with low volatility^32^. Their influence is therefore negligible compared to the influence of the hydrocarbons that remain on the substrate (including at the entrance). This relation between entering probability and chemical marking leads to positive feedback that affects the difference between the probabilities of entering the DS and WS. Once inside, RH had no influence on the decision to leave of the isolated individuals, as shown by the analysis of time-bouts of them (2 A). At the group level, the individual probability of leaving decreased with the sheltered population. However, in this case RH did influenced the leaving probability: this decrease was less marked in the wet shelter (Figure S2) strongly suggesting that the influences of conspecifics is weaker in the WS. The nonlinear least square fitting of the leaving probabilities (Table 2) demonstrated that differences between the leaving probabilities resulted from modulation of the strength of social interactions (*γ*_*w*_ < *γ*_*d*_) by RH. We acknowledge that our model does not provide any information on how this modulation takes place and that different hypotheses can be formulated. However, the literature^7,29,38^ allows us to exclude competition between water and hydrocarbons at the level of receptors on the antennae. Moreover, to the best of our knowledge, there are no data showing that humidity decreases pheromone marking during the span of time used in our experiments. Therefore, we believe that the threshold response to conspecific pheromones might differ among levels of HR^24,39,40^. Indeed, for cockroaches, the aggregates formed under high levels of humidity are less dense (as shown by Dambach and Goehlen^15^) and therefore less stable, thereby leading to an increase in the leaving probability. This decrease in density is in agreement with the mechanisms of water regulation. High levels of humidity and gregariousness contribute to reducing water loss^11,14,16,17^. In fact, a classical geometric hypothesis is put forth to explain the individual reduction of water loss in an aggregate. The water loss rate is proportional to the surface area exposed, which scales to the power 2/3 of the number of aggregated individuals, and the initial body water content is proportional to this number. Thus, water loss per individual is proportional to (*N*_individuals_^*−*2/3^). A dense aggregate may also create a locally humid microclimate for all individuals in a small volume (Schliebe, 1988, cited by Broly^17^). Conversely, it has been suggested that a high humidity level increases horizontal transmissions of pathogens^41^ and favours fungal development^42,43^. At high densities, such transmission is evidently facilitated. The inversion of shelter selection between isolated individuals and groups might be seen as an efficient physiological and prophylactic strategy in response to humidity. Furthermore, cuticular hydrocarbons play a complex role in these collective behaviours^44^, as they also participate in resistance to desiccation^36,45^ and constitute a barrier against pathogens^46^. Tackling these bio-physico-chemical mechanisms and their relation to collective behaviour quantitatively would help disentangle the distal causes of the inversion of the preferences for a resting place between isolated individuals and the groups observed in this study. Finally, we shed light on the mechanisms behind this counter-intuitive collective phenomenon that may be generic and present in many species where the same biotic or abiotic environmental factor affects the strength of social interactions^5,16,27,28,47–49^.

## Methods and Materials

### Biological model and experimental setup

Cockroaches, *Periplaneta americana* (Dictyoptera: Blattidae; Linnaeus), were obtained from strains reared in breeding facilities (five Plexiglas vivaria of 80 × 40 × 100 *cm* (WxLxH)) of the Université Libre de Bruxelles. Each vivarium contained approximately 1000 individuals of both sexes and at all developmental stages, and we provided dog pellets and water twice a week. The cockroaches used in this study measured from 35 to 50 mm in length.

### Experimental setup

Experiments were carried out on 54 isolated individuals and 29 groups of 16 adult males of *P. americana* without external damage to exclude any behavioural variation linked to the ovarian cycle. Each trail lasted 10800 *s*. The experimental setup (Fig. 3B) included a circular arena covered with a paper layer (120 *g*/*m*^2^), surrounded by a polyethylene ring (diameter: 100 cm, height: 20 cm) and with a light source (5 M Ustellar Dimmable Kit Ruban Led, 2835 SMD Led, white cold 6000 K) placed above the setup to provide a homogeneous light intensity of 415 lux at ground level. To avoid any visual cues, the arena was placed inside a white box of 10.5 × 13.0 × 10.5 *cm* (W × L × H). Two shelters (one dry, one wet; see below) made of transparent Plexiglas pipes (H: 30 cm; D: 15 cm) were covered with red-coloured filter film (Rosco E-color 19: fire), and their upper surface was covered with a black carton, allowing a light intensity inside the shelter of 22 lux. A transparent ceiling was placed at 2.5 cm to reduce the volume of the shelter. The centre of each shelter was located 23 cm from the edge of the arena. Shelters had two symmetrically opposed entrances of 2 × 1.5 cm (W × H) aligned to the centre of the arena. To control humidity inside the shelter, a hole of 11 cm covered with a plastic grid was made in the floor of each shelter. The floor of the arena was covered with a paper layer, with two openings at the locations of the shelters. This paper was changed, and the shelters, including the floor under each shelter, were cleaned after every trial to avoid chemical marking. Each shelter was large enough to accommodate at least 16 cockroaches.

### Humidity control

Cockroaches are photophobic; therefore, both shelters are perceived as resting sites during the diurnal phase^29^. Moreover, cockroaches are sensitive to the “wetness” of the environment, which is reflected by the saturation deficit^50^, which depends on temperature as well as RH. Because all the trials were conducted at 25 × 2, the saturation deficit depend only on RH. The “dry” shelter had the same RH as the experimental room, which was maintained at 42.5 ± 7 %. The wet shelter had an RH of 92 ± 5 %, generated by adding 40 ml of tap water^15^ to a Petri dish, which was placed 10 cm beneath the plastic grid. The room conditions were measured with a multi-function climate-measuring instrument (Testo 435 coupled to a temperature and humidity probe). The humidity and temperatures under the shelter were measured during all the experiments with humidity and temperature sensors (DHT22) beneath the plastic grid on the floor of the shelter.

### Data and statistical analysis

Sheltered individuals were recorded by a video camera (17 frames/*s*) (Logitech webcam C920 HD 1080p) located on the upper side of each shelter. The time in second (precision of 0.2 *s*) of an entry/exit as well as the number of sheltered cockroaches were encoded using Solomon coder https://solomoncoder.com/) for isolated individuals and VlC media player for the groups.

Data analysis, statistical tests and simulations were performed using R software (R Core Team 2018, R Foundation for Statistical Computing, https://www.r-project.org/) and Python (Python Software Foundation. Python Language Reference, version 2.7.15 at http://www.python.org). The significance of the statistical tests was fixed to an *α* = 0.05^51^. *χ*^2^ tests were used to compare the total sheltered population size between the isolate and group trials, the number of sheltered individuals between the DS and WS, the numbers of entries of the DS and WS and the numbers of wins (summing all trials with most of the individuals choosing the WS (DS)). A permutation^52^ test was used to compare the distributions of the populations under each shelter type, the number of wins of each shelter and the number of entries of each shelter for the isolate (1000 × 54 realisations) and group (1000 × 29 realisations) trials. For the groups, we tested whether the observed distribution of sheltered individuals in the DS was compatible with a non-social situation, where individuals lack social interactions, or in other words, whether it followed a binomial distribution^53^. For this purpose, we computed the theoretical variance of the total sheltered population size expected for individuals without interactions. We thus performed simulations (1000 realisations) considering that the individual mean probability of sheltering was equal to the proportion of the total population in the shelter (sheltered individuals/population size) at the end of experiments. We used Wilcoxon rank sum and signed rank tests to compare (1) the mean number and the proportion of entries per individual and per trial between individual and group conditions; (2) the distribution of the time-bouts between the DS and WS; and (3) the ratio of total entries/total exits per trial between the DS and WS for the individuals and groups. Moreover, we used survival analysis (log-rank test) of the time-bouts spent under each shelter to determine the influence of RH on the probability of leaving per unit time. Finally, a permutation test of the number of wins was performed every 600 *s* for both the isolate and group trials.

## Supplementary information

Supplementary Information

## Data Availability

Our data are available from the figshare repository: 10.6084/m9.figshare.8313569.v1.
