# Supplementary material for: Conflictual influence of humidity during shelter selection of the American cockroach (Periplaneta americana)
Source: Sci Rep. 2019 Dec 30;9:20331. doi: 10.1038/s41598-019-56504-w (PMC6937323; doi:10.1038/s41598-019-56504-w)
Supplement: Supplementary file 1 — Supplementary Information [file 41598_2019_56504_MOESM1_ESM.pdf]

Conflictual influence of humidity during shelter selection of the American cockroach (*Periplaneta americana*)

Mariano Calvo Martín[1,2*], Stamatios C. Nicolis[1], Isaac Planas-Sitjà[3], and Jean-Louis Deneubourg[1]

[1]Biological and Artificial Self-organised Systems Team, Université Libre de Bruxelles, Brussels, Belgium
[2]Evolutionary Biology & Ecology Unit, Université Libre de Bruxelles, Brussels, Belgium
[3]Systematic Zoology Laboratory, Tokyo Metropolitan University, 1-1 Minami-Osawa, Hachioji, Tokyo, 192-0397, Japan
[*]mcalvoma@ulb.ac.be

Differences between leaving rates from the shelters

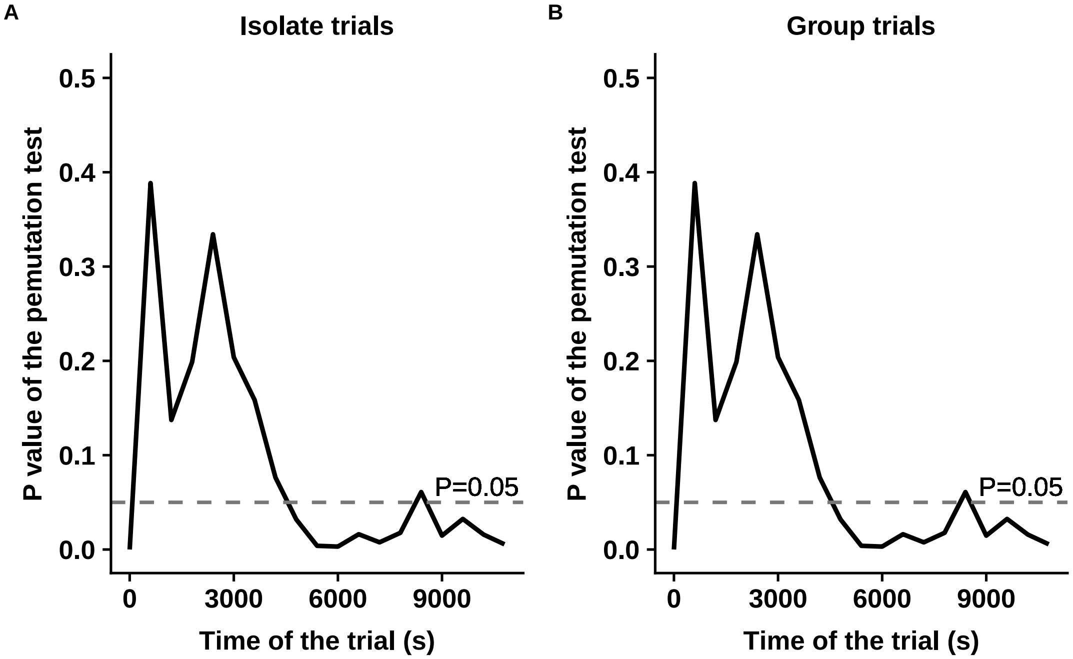

Figure S1. P values of permutations tests of the number of wins of a shelter over time. The dashed horizontal line indicates statistical significance at α =0.05. (A) Wins of the WS for the isolate trials. (B) Wins of the DS for the group trials.

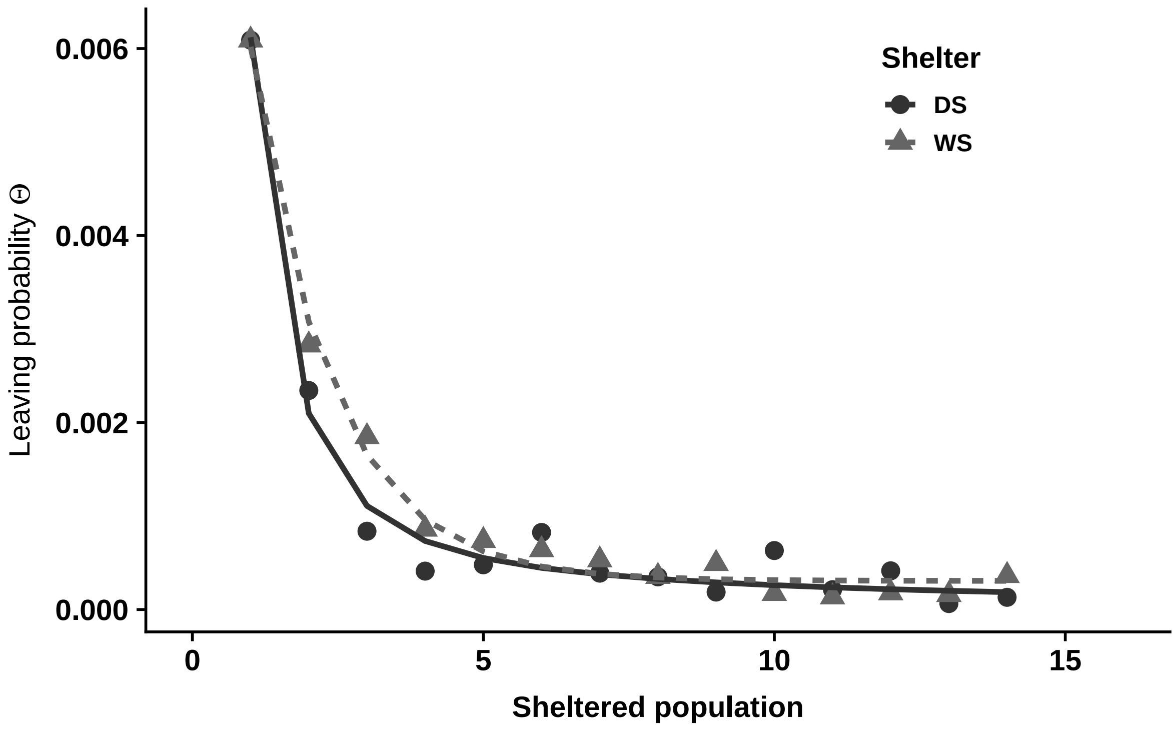

Figure S2. Probability of leaving the shelter as a function of the number of sheltered individuals in the DS (dark grey dot) and the WS (light grey dot) and their respective fitted values from nonlinear least square fitting (DS: dark grey dashed line and WS: light grey dashed line) (for fitted parameters, see table 2.

Differences between leaving rates from the shelters

Being understand that $\varepsilon_w = \varepsilon_d$ and $\beta_w = \beta_d$ the difference between individual leaving rates from the DW and the DS (eq. 11) is:

$$\Delta\Theta\,(i_d) = \varepsilon(\; e^{-\gamma_w(i_w-1)} - \; e^{-\gamma_d(i_d-1)}) \tag{S1}$$

Eq. S1 displays a maximum at:

$$i = 1 + \frac{\ln\frac{\gamma_w}{\gamma_d}}{\gamma_w - \gamma d} \tag{S2}$$

Figure S3 shows this difference as a function of the sheltered individuals for the values of γ_w and γ_d. The largest differences between the leaving rates from WS and DS are between 2 and 6 sheltered individuals. For larger sheltered population these differences become negligible. This is true for the values $\gamma_w(\gamma_d)$ estimated from the non-linear fitting and from the simulation (see table 2 and section model).

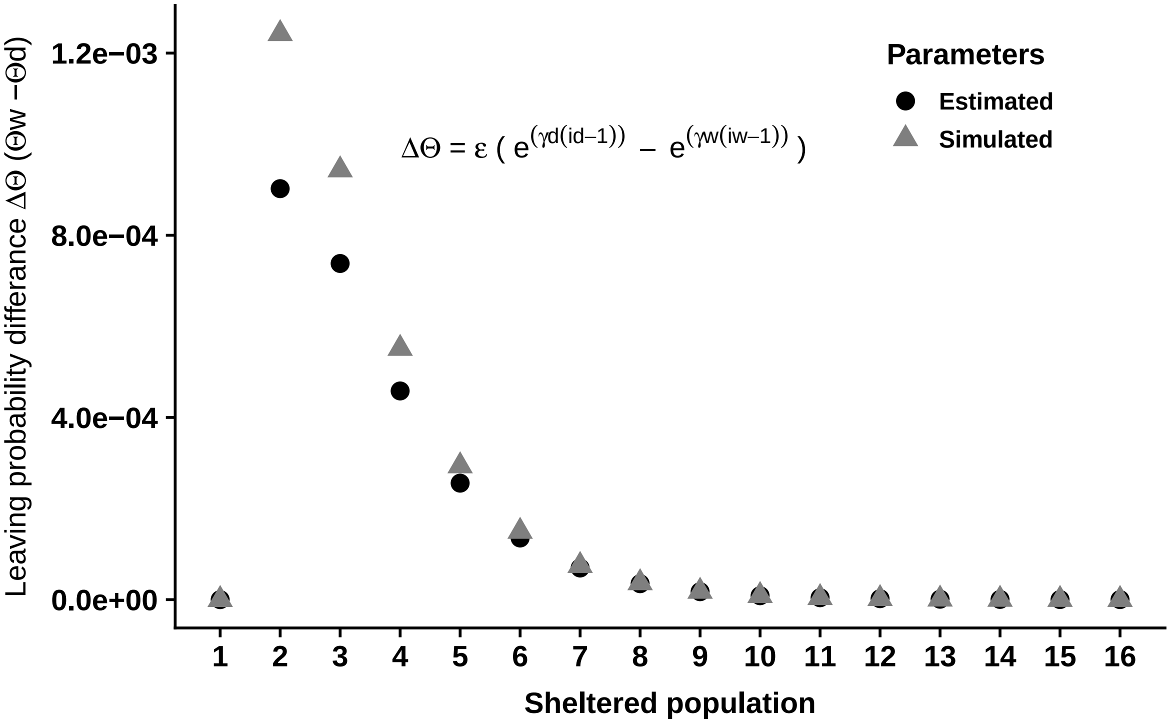

Figure S3. Difference of leaving probabilities $\Delta\Theta$ ($\Theta_w - \Theta_d$) as a function of the number of sheltered individuals for the estimated parameters from table 2 (black dot, $\varepsilon = 5.8 \times 10^{-3}$; $\gamma_d = 1.051$; $\gamma_w = 0.72$) and for the simulated values (light grey triangle, $\varepsilon = 5.8 \times 10^{-3}$; $\gamma_d = 1.3$; $\gamma_w = 0.72$).
